# Activation of D1R/PKA/mTOR signaling cascade in medial prefrontal cortex underlying the antidepressant effects of *l*-SPD

**DOI:** 10.1038/s41598-017-03680-2

**Published:** 2017-06-19

**Authors:** Bing Zhang, Fei Guo, Yuqin Ma, Yingcai Song, Rong Lin, Fu-Yi Shen, Guo-Zhang Jin, Yang Li, Zhi-Qiang Liu

**Affiliations:** 1 Key Laboratory of Receptor Research, Shanghai Institute of Materia Medica, Chinese Academy of Sciences, Shanghai, 201203 China; 2 University of Chinese Academy of Sciences, No. 19A Yuquan Road, Beijing, 100049 China; 3 Department of Anesthesiology, Shanghai First Maternity and Infant Hospital, Tongji University School of Medicine, Shanghai, China

## Abstract

Major depressive disorder (MDD) is a common neuropsychiatric disorder characterized by diverse symptoms. Although several antidepressants can influence dopamine system in the medial prefrontal cortex (mPFC), but the role of D1R or D2R subtypes of dopamine receptor during anti-depression process is still vague in PFC region. To address this question, we investigate the antidepressant effect of levo-stepholidine (*l*-SPD), an antipsychotic medication with unique pharmacological profile of D1R agonism and D2R antagonism, and clarified its molecular mechanisms in the mPFC. Our results showed that *l*-SPD exerted antidepressant-like effects on the Sprague-Dawley rat CMS model of depression. Mechanism studies revealed that *l*-SPD worked as a specific D1R agonist, rather than D2 antagonist, to activate downstream signaling of PKA/mTOR pathway, which resulted in increasing synaptogenesis-related proteins, such as PSD 95 and synapsin I. In addition, *l*-SPD triggered long-term synaptic potentiation (LTP) in the mPFC, which was blocked by the inhibition of D1R, PKA, and mTOR, supporting that selective activation of D1R enhanced excitatory synaptic transduction in PFC. Our findings suggest a critical role of D1R/PKA/mTOR signaling cascade in the mPFC during the *l*-SPD mediated antidepressant process, which may also provide new insights into the role of mesocortical dopaminergic system in antidepressant effects.

## Introduction

Major depressive disorder (MDD) is a common neuropsychiatric disorder characterized by low mood, decreased pleasure, feelings of despair, loss of motivation and anhedonia, and is one of the leading causes of total disability worldwide^1, 2^. Currently, available antidepressants, such as selective serotonin reuptake inhibitors (SSRIs), are prescribed with limited efficacy, including a delayed response onset of weeks or months, partial responsiveness or non-responsiveness, and some serious side effects^3, 4^. Most of the current antidepressant medications, such as selective norepinephrine reuptake inhibitors (SNRIs) and SSRIs, target the monoamine systems and result in elevation of the norepinephrine and serotonin levels in the synaptic cleft^5, 6^. Truly novel treatments are still needed to address these limitations and improve antidepressant therapeutics.

The mesocorticolimbic DA system has been shown to be involved in emotion-related behaviors, especial in the encoding of stressful events^7, 8^. The medial prefrontal cortex (mPFC) receives ventral tegmental (VTA) originated DA neurons and modulates executive control, cognition and social behaviors, which are believed to associate with psychiatric diseases. Note that dopamine receptors subtypes in a specific brain region may play diverse functions in the pathogenesis and pharmacological treatments of such diseases. For example, evidence from anatomical studies showed that the density and distribution of D1-like receptors (D1R) in the mPFC are much higher (more than 20-fold) than D2-like receptors (D2R). Additionally, D1R is mostly located on the dendrites and spines of pyramidal cells^9–12^, which predicts that D1R-mediated mPFC excitability may dominate the emotional-related behaviors of mesocorticolimbic DA system. Indeed, preclinical studies have emphasized the crucial role of D1 agonism in pharmacological mechanism of depressant. Bupropion and nomifensine, which are dopamine re-uptake inhibitors, significantly decreased the duration of immobility^13^. Direct evidence also showed a robust antidepressant-like effect by the selective dopamine D1R agonist^14, 15^. Moreover, the long-term potentiation (LTP) induced by the D1R activation in the mPFC may contribute to improved cognitive function^16, 17^. In schizophrenia disease, the negative and cognitive symptoms can be significantly ameliorated by the D1R agonist^10, 18, 19^.

**Figure 1:**
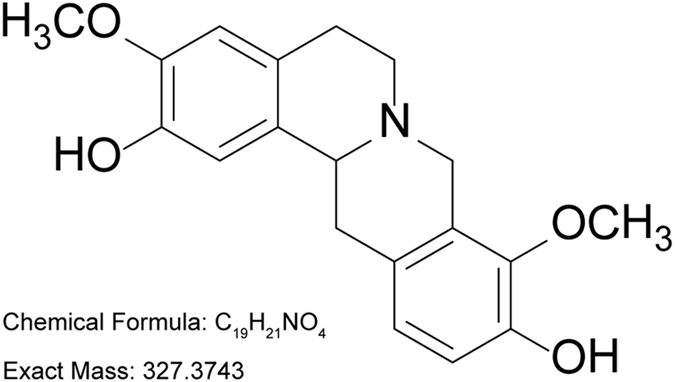
The chemical structure of *l*-SPD.

Although many studies have provided evidences of dopaminergic-mediated regulation in emotion-related behaviors, the molecular mechanism of its antidepressant-like effect is less known. In the present study, levo-stepholidine (*l*-SPD, Fig. 1), prescribed as an antipsychotic medication with D1R agonist/D2R antagonist pharmacological profile, was used to investigate the molecular mechanisms of the mPFC during antidepressant treatment. *l*-SPD is a tetrahydroberberine alkaloid obtained from the Chinese herb *Stephania intermedia*, which has shown high affinity for D1-like receptors and modest affinity for D2-like receptors^20–23^. Preclinical and clinical studies had shown that *l*-SPD could relieve both positive and negative symptoms of schizophrenia mediated by D2R antagonism in the nucleus accumbens (NAc) and D1R agonism in the mPFC, respectively^20, 24^. As the improvement of negative and cognitive symptoms mediated by D1R agonism in schizophrenia, D1R agonism may share similar pharmacological action with depressive-related dysfunction in the mPFC. Previous studies also suggested D1R agonism enhanced the dopaminergenic transmission in the mPFC and improved cognition and motivation in schizophrenia and depression^25, 26^. Therefore, we hypothesize that *l*-SPD may also exert antidepressant effects by reversing deficiency in cognitive functions.

In this work, we found that *l*-SPD had a fast antidepressant effect on Sprague-Dawley rat chronic unpredictable mild stress (CMS) model of depression, which is a valid animal depressive model widely used to screen potential antidepressant candidates. Mechanism investigations reveal that *l*-SPD worked as a specific D1R agonist in the PFC to activate downstream signaling of PKA/mTOR intracellular signaling pathway, which in turn produced an increase of synaptic proteins and further triggers the long-term enhancement of synaptic neurotransmission. This process of D1R mediated signal transduction may be the underlying mechanism of fast antidepressant effect of *l*-SPD.

## Results

### Antidepressant effect of *l*-SPD on Sprague-Dawley rat CMS model of depression

**Figure 2:**
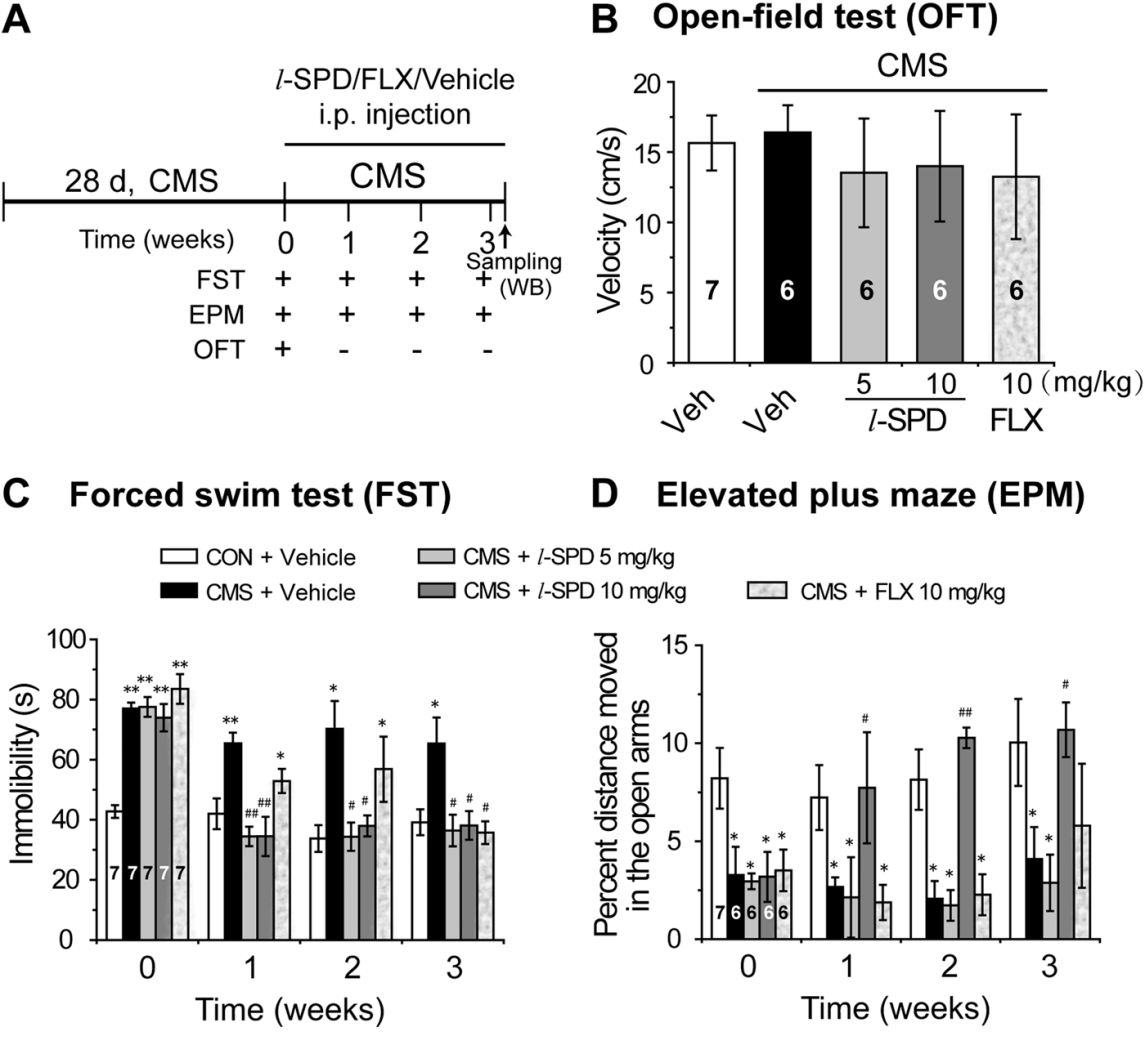
*l*-SPD reverses depressive-like behaviors in CMS Sprague-Dawley rat model of depression. (**A**) The schematic representation for experimental procedures. The rats were exposed to CMS stressors daily for 28 days. Then, the rats were treated with *l*-SPD (5 mg·kg^−1^ and 10 mg·kg^−1^), fluoxetine (10 mg·kg^−1^) and vehicle (solvent control) once per day for 3 weeks. During the drug administration, the behavioral tests were performed once per week and the CMS stimulations were continued. The mPFC tissues were obtained 24 h after the last behavioral tests completed. (**B**) Open field test were performed at week 0 and the velocity of rats were measured. (**C**) Forced swim tests were performed at week 0, 1, 2 and 3 and the immobility time of rats were measured. (**D**) Elevated plus maze was performed at week 0, 1, 2 and 3, and the percent distance moved by rats in the open arms were measured. The inserted numbers represent the number of animals in each experimental group. *p < 0.05, **p < 0.01, compared to the vehicle treatment. ^#^ *p* < 0.05, ^##^ *p* < 0.01, compared to the CMS group.

We initially examined whether *l*-SPD would have a therapeutic effect on an animal model of depression. The chronic mild stress (CMS) model is a valid and common animal model for screening antidepressant candidates and studying the mechanism of pathological depression^27–29^. Here, the Sprague-Dawley rat CMS depressive model was used to evaluate the anti-depressive effect of *l*-SPD. After 4-weeks of CMS, approximately 70% of the stressed rats had depressive- and anxiety-like behaviors based on the forced swimming^30^ and elevated plus maze tests^31^, respectively. All depressive- and anxiety-like animals were then randomly divided into four groups and treated with vehicle, 10 mg·kg^−1^ fluoxetine, 5 mg·kg^−1^ and 10 mg·kg^−1^ *l*-SPD (Fig. 2A). The naive and aged-matched animals were also treated with vehicle. As shown in Fig. 2B, the velocity showed no significant differences within the five groups in the open field test at week 0, which excluded the possibility of psychomotor substances that may elicit false positive results^32, 33^. The depressive-like behavior was examined by forced swimming, and the results showed that *l*-SPD-treated animals significantly reversed the abnormal of CMS-induced immobility with just one week of administration; however, this reversal course required 3 weeks with fluoxetine treatment compared with vehicle-treated animals (Fig. 2C). Similarly, for the elevated plus maze test, the reversal of the percent time in open arms only required 1 week of *l*-SPD administration, however, even 3-week fluoxetine administration could not reach the same reversal (Fig. 2D). The repeated treatments of *l*-SPD were also administrated in naïve C57BL/6 mice, and the antidepressant- and anxiolytic-like behaviors were observed with 10-day administration once daily (Figure S1A–C in supplemental results). These results suggested that the depressive-like phenotype induced by CMS could be normalized by the chronic administration of *l*-SPD; furthermore, *l*-SPD had relatively faster therapeutic onset than the SSRI fluoxetine.

### *l*-SPD enhanced synaptic function by the activation of mTOR signaling in the mPFC

**Figure 3:**
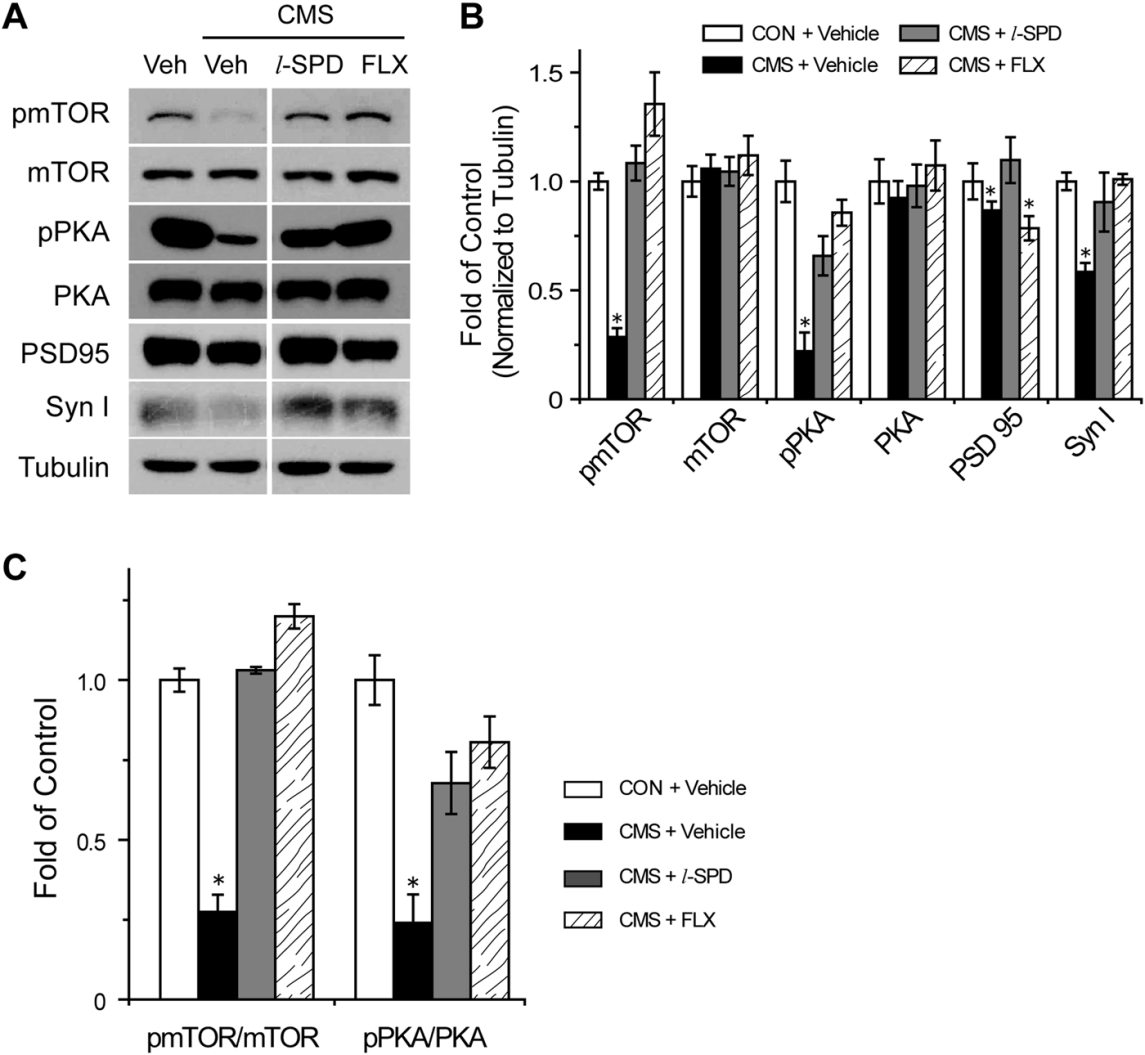
*l*-SPD enhances synaptic function by the activation of mTOR signaling in the mPFC. (**A**) Western blot images and (**B**) quantification analysis of pmTOR (Ser 2448), mTOR, pPKA (Thr 197), PKA, PSD 95, synapsin I in mPFC of CMS-induced rats or control rats with 3-week *l*-SPD (5 mg·kg^−1^ or 10 mg·kg^−1^), fluoxetine (10 mg·kg^−1^) and vehicle treatment group (solvent control) administration. The β-tubulin was determined as reference protein. (**C**) Quantification analysis of pmTOR/mTOR and pPKA/PKA ratios in mPFC and normalized to that of control group (without CMS treatment). *p < 0.05, compared to the vehicle treatment group (CON + Vehicle).

To examine the signaling pathway involved in the antidepressant effect of *l*-SPD, mPFC tissues were obtained 24 hours after behavioral tests. Changes in protein levels were then analyzed by western blot. Recent studies have shown that the synaptic structures were quickly remodeled by mTOR signaling within 2 h after ketamine administration^34, 35^. The regulation of mTOR signaling have been demonstrated to associate with fast antidepressant effects. 24 h after the behavioral test, the mPFC brain tissues of vehicle-, fluoxetine- and *l*-SPD-treated CMS groups were obtained for western blotting analysis. As shown in Fig. 3A–C, western-blotting results showed that CMS indeed resulted in decreased levels of pmTOR (Ser 2448) and pPKA (Thr 197), which could be reversed by chronic administration of *l*-SPD (10 mg·kg^−1^, i.p.) and fluoxetine (10 mg·kg^−1^, i.p.). The total expression level of mTOR and PKA showed no changes between groups, therefore, as like pmTOR (Ser 2448) and pPKA (Thr 197), the pmTOR (Ser 2448)/mTOR and pPKA (Thr 197)/PKA ratios were decreased by CMS stimulation and reversed by *l*-SPD or fluoxetine treatment also. As the activation of mTOR has been functionally linked with protein synthesis in the synapse^36^, we further determined whether the treatment by *l*-SPD affected the expression of synaptogenesis-related proteins. We analyzed the expression level of presynaptic protein synapsin I and postsynaptic protein PSD 95 in the mPFC. Our results demonstrated that *l*-PSD administration reversed the low level of both proteins induced by CMS (Fig. 3A and B). We also observed the increasing levels of pmTOR (Ser 2448) and PSD 95 in the naïve C57BL/6 mice administrated by *l*-SPD for 10 days (Fig. S2A–D in supplement). These results indicated that *l*-SPD enhanced mTOR signaling and consequently promoted synaptogenesis in the mPFC, which may be associated with the antidepressant-like effect of *l*-SPD.

### *l*-SPD selectively activated mTOR signaling by D1R agonism

**Figure 4:**
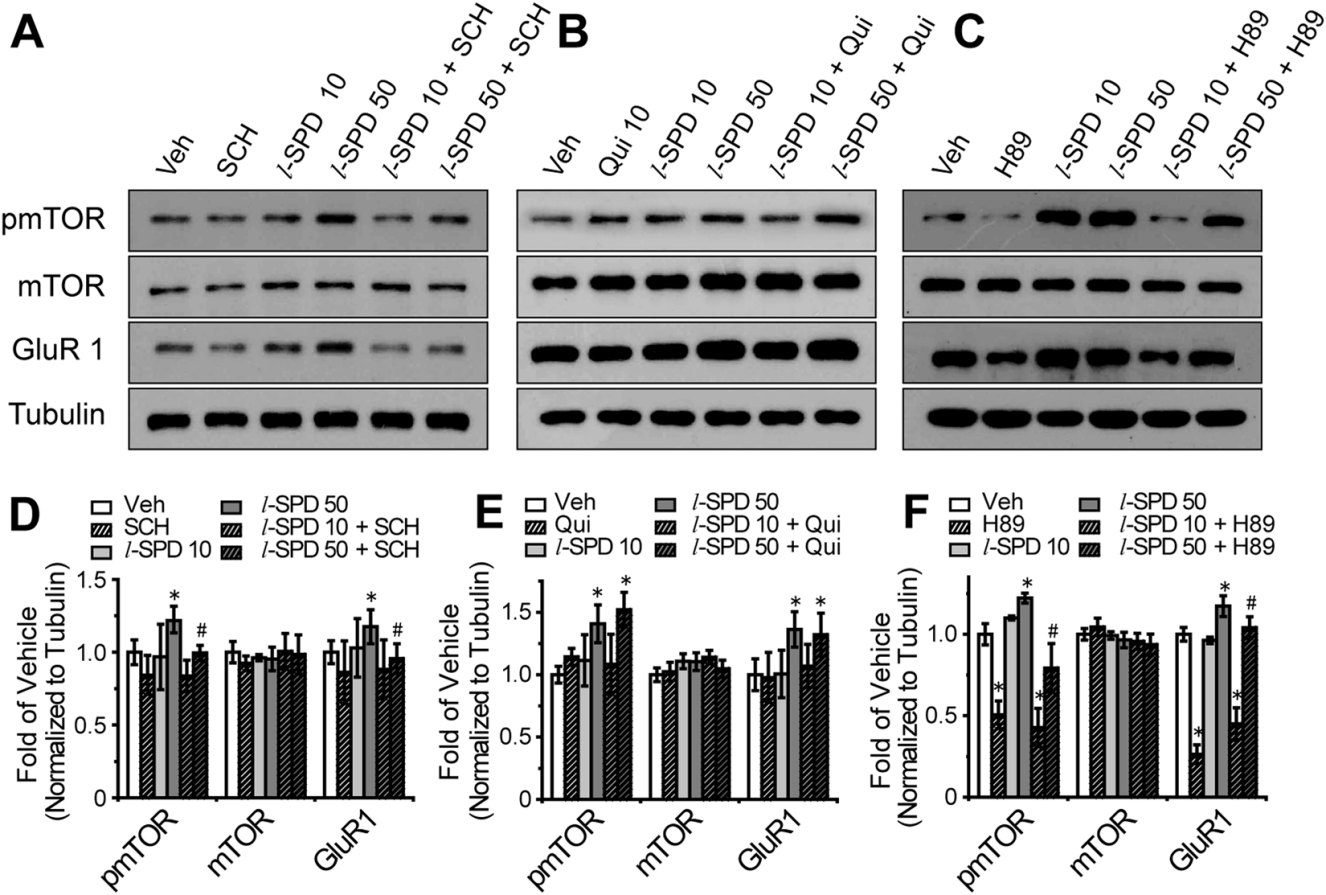
*l*-SPD selectively activates mTOR signaling by D1R agonism rather than D2R antagonism. One week cultured primary cortical neurons were incubated with drugs for 4 hours. (**A**) Western blot images and (**D**) quantification analysis of pmTOR (Ser 2448), mTOR, GluR1 in the treatment of vehicle, *l*-SPD (10 µM or 50 µM), SCH23390 (10 µM) and SCH23390 (10 µM) with *l*-SPD (10 µM or 50 µM). (**B**) Western blot images and (**E**) quantification analysis of pmTOR (Ser 2448), mTOR, GluR1 in the treatment of vehicle, *l*-SPD (10 µM or 50 µM), Quinpirole (10 µM) and Quinpirole (10 µM) with *l*-SPD (10 µM or 50 µM). (**C**) Western blot images and (**F**) quantification analysis of pmTOR (Ser 2448), mTOR, GluR1 in the treatment of vehicle, *l*-SPD (10 µM or 50 µM), H89 (10 µM) and H89 (10 µM) with *l*-SPD (10 µM or 50 µM). The β-tubulin were determined as reference protein. *p < 0.05, compared to the vehicle treatment group. ^#^ *p* < 0.05, compared to the 50 µM *l*-SPD treatment group.

Due to *l*-SPD functioning not only as a D1R agonist but also as a D2R antagonist, we further determined whether D1- or D2-subtype of dopamine receptor mediated the enhancement of mTOR signaling by administration of *l*-SPD. Cultured neurons in the primary cortex, obtained from embryos of Sprague–Dawley rats, were incubated with drugs for 4 hours. The initial result confirmed that the pmTOR (Ser 2448) level significantly increased by the treatment of 50 µM *l*-SPD compared to the vehicle-treated group (Fig. 4A,D). Furthermore, the enhancement of pmTOR (Ser 2448) was blocked by the D1R antagonist (SCH 23390) but not by the D2R agonist (quipirole) (Fig. 4A,B,D,E). The total mTOR level showed no changes to *l*-SPD, SCH 23390 and quipirole (Fig. 4A,B,D,E). We also found that the expression of GluR1, a subunit of AMPA receptor, was elevated by the incubation of *l*-SPD alone and blocked by SCH 23390 rather than quipirole (Fig. 4A,B,D,E). These results suggested that *l*-SPD enhanced mTOR signaling and elevated the expression level of GluR1 by activation of D1R in the primary cortical neurons.

Protein kinase A (PKA), a cAMP-dependent protein kinase, can be regulated by the activation or deactivation of dopamine receptors. We therefore examined the role of PKA in the D1R-induced activation of mTOR signaling. In accordance with the above experiments in culture neurons, we inhibited the activation of PKA by H89. Our results showed that the phosphorylation level of pmTOR (Ser 2448) and expression level of GluR1 was significantly decreased by the treatment of H89 (total mTOR expression level showed no changes). Moreover, the *l*-SPD-induced increased levels of pmTOR (Ser 2448) and GluR1 were also neutralized by PKA inhibition (Fig. 4C,F). These results further suggested that the *l*-SPD-induced activation of mTOR signaling may require D1 agonism and the involvement of PKA activation.

### l-SPD selectively triggered LTP by D1R agonism relying on mTOR activation

**Figure 5:**
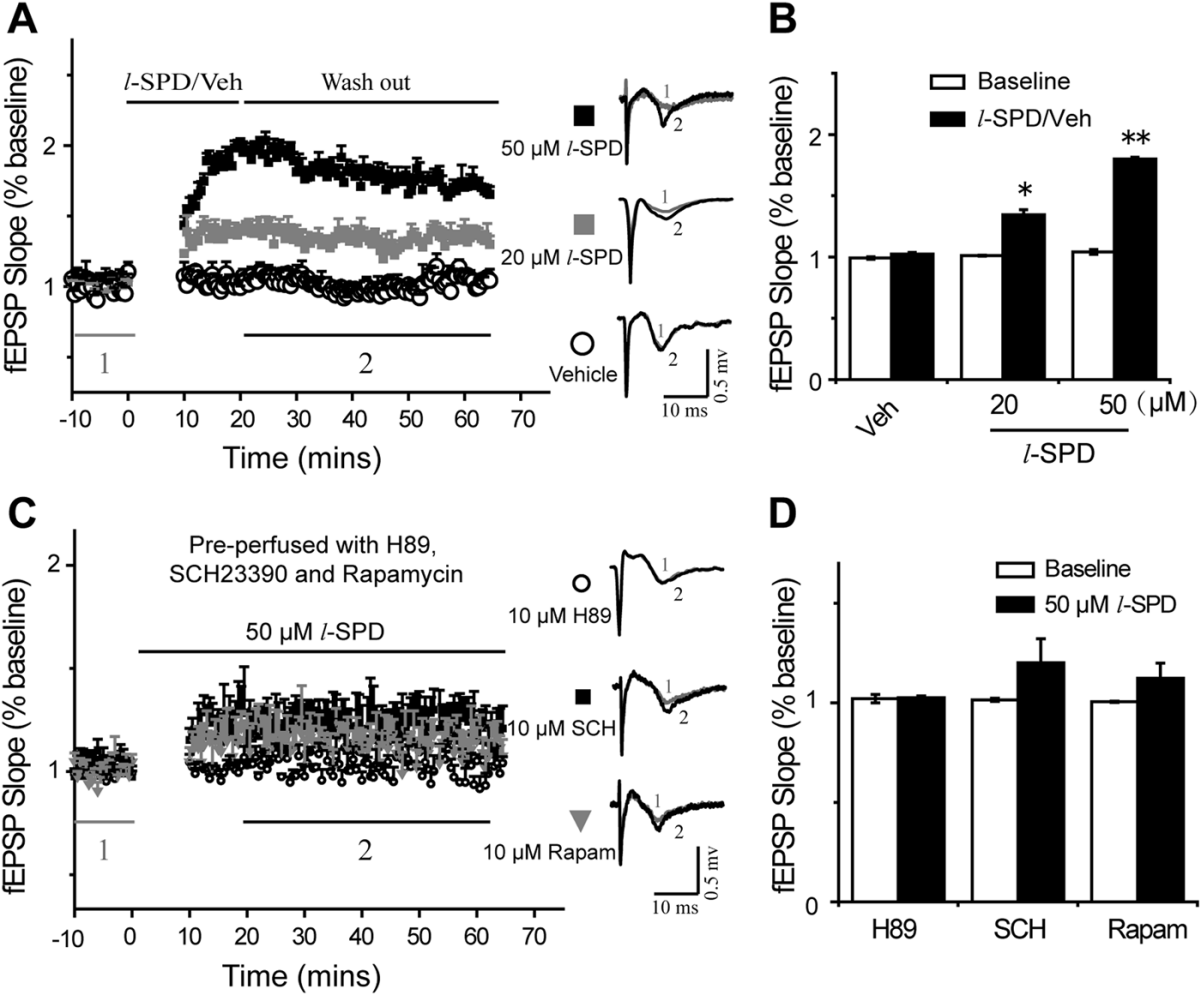
*l*-SPD triggered LTP in mPFC, and LTP was blocked by D1R antagonist (SCH23390), PKA inhibitor (H89) and mTOR inhibitor (rapamycin). (**A**) The baseline of field EPSP (fEPSP) was recorded for 10 minutes, followed by *l*-SPD (20 µM or 50 µM) and vehicle treatment for 10 minutes. Initial fEPSPs were plotted as a function of time. The inserted waveforms on the right side represent samples from baseline (showed in inserted digit 1) and wash-out recording (shown in inserted digit 2). (**B**) Summary bar graph showing the effects of *l*-SPD treatments on the fEPSP slope. (**C**) The brain slices were pre-perfused with SCH23390, H89 and rapamycin at a concentration of 10 µM for 30 minutes. The next processes were similar to the procedures described in (**A**). The whole recording time lasted more than 75 minutes. The representative traces of baseline (marked 1) and *l*-SPD treated recording (marked 2) are shown at right. (**D**) The effects of *l*-SPD (50 µM) on fEPSP slope with D1R, PKA or mTOR inhibition. *p < 0.05, **p < 0.01, compared to baseline.

As the critical role of mTOR signaling is in the maturation of synaptic structure and function, we next performed the field excitatory postsynaptic potentiates (fEPSP) experiments to examine whether *l*-SPD affected synaptic transmission in the mPFC. The results showed that the slope of fEPSP could be significantly enhanced by incubation of *l*-SPD within 10–20 minutes, and the enhancement lasted more than 45 minutes after *l*-SPD was washed out. Furthermore, the enhancement of the fEPSP slope was concentration-dependent; the concentration at 50 µM showed a stronger effect than at 20 µM (Fig. 5A,B). These results suggested that *l*-SPD strengthened the postsynaptic transmission in the mPFC.

Having shown that *l*-SPD-induced activation of mTOR signaling may require D1R agonism induced PKA activation (Fig. 4), to further identify the role of D1R, PKA and mTOR in the effect of *l*-SPD strengthened postsynaptic transmission in the mPFC, we pre-treated mPFC slices with D1R antagonist (SCH 23390, 10 µM), PKA inhibitor (H89, 10 µM) and selective mTOR inhibitor (rapamycin, 10 µM) for 30 minutes and then performed the fEPSP experiments as described above. The results showed that *l*-SPD failed to enhance the slope of fEPSP after pre-treatment with SCH 23390, H89 and rapamycin in the mPFC (Fig. 5C,D). These results suggested that the *l*-SPD strengthened postsynaptic transmission was mediated by the activation of D1R/PKA/mTOR signaling pathway in the mPFC.

## Discussion

*l*-SPD is a D1R agonist and D2R antagonist, prescribed as a new generation of anti-schizophrenia drug, which showed remission for both positive and negative symptoms of schizophrenia^37^. In the present study, we found that *l*-SPD had antidepressant- and anxiolytic-like effects with relatively fast therapeutic onset (Fig. 2). To further understand the underlying molecular mechanism, we investigated the mTOR signaling pathway and the expression levels of synaptogenesis-related proteins, such as PSD 95, GluR1 and synapsin I. Our results demonstrated that the administration of *l*-SPD resulted in increasing level of pmTOR (Ser 2448) synaptogenesis-related proteins in the mPFC (Fig. 3 and Figure S2). This, in turn, functionally produced a long-term enhancement (LTP) of synaptic plasticity recorded by the method of electrophysiology *in vitro* (Fig. 5). Conversely, the increased level of pmTOR (Ser 2448)/synaptic-related protein by *l*-SPD was blocked by D1R/PKA inhibition rather than D2R activation (Fig. 4). Furthermore, the *l*-SPD induced LTP in the mPFC was blocked by the inhibition of D1R, PKA and mTOR (Fig. 5), which further indicated that D1R-dependent activation of PKA/mTOR signaling may be the primary mechanism for the *l*-SPD-induced antidepressant- and anxiolytic-like effects.

The medial prefrontal cortex (mPFC) regulates decision making, thought, actions and emotion through extensive connections with other brain regions. The mPFC is also responsible for working memory, which is the ability to keep current events in mind or bring back information from long-term storage and integrate the relevant representations. However, the mPFC is also a brain region that is vulnerable to stress. Even short and mild stressors can cause rapid dendritic shrinkage and dramatic prefrontal cognitive disabilities^38, 39^.

The dopaminergic system is known to be critical to higher cognition and reward. Dysfunction of the mesocortical dopaminergic system is proposed to be involved in the pathophysiology of depression^26, 40^. Previous studies have revealed that the concentration of DA metabolites in cerebrospinal fluid is lower in depressed patients^41, 42^. Studies on behavior models have also demonstrated impaired DA release in presynaptic, decreased D1R density in the PFC and decreased burst firing rate in VTA-mPFC DA neurons^43–45^. Therefore, the diminished dopaminergic transmission is correlated to the pathophysiology of depression. Dopamine receptors are divided into two groups: D1-like receptors (D1R) and D2-like receptors (D2R). Activation of D1R tends to strengthen synaptic transmissions^46, 47^. The study by Paolo S. D’Aquila *et al*. showed that D1R agonists exert antidepressant-like effects in animals and proposed that D1R agonism may be the potential target for screening antidepressant drugs^15^. *l*-SPD is a prescribed medication that targets the dopaminergic system^48^. Functional studies revealed that *l*-SPD is D1R agonist and D2R antagonist^23, 49^, and enhanced synaptic plasticity in hippocampus via activating D1R/PKA signaling pathway^50^. In this study, we confirmed that *l*-SPD exerted antidepressant effects through D1R agonism in the mPFC. Our mechanism studies revealed that the level of synaptogenesis-related proteins, such as GluR1, synapsin I and PSD 95 were increased by *l*-SPD administration. Altogether, these findings suggested that D1R agonism of *l*-SPD exerted an enhancement of cortical dopaminergic transmission, resulting in enhancement of the synaptic plasticity in the mPFC, and further improving the cognitive function, which may be the underlying mechanism of *l*-SPD-evoked antidepressant action.

Multiple reports support the role of dopamine in the pathophysiology of MDD^40^. The distribution of D1 family of dopamine receptors is at least 20-fold more abundant than D2 family receptors in the mPFC^10^. Furthermore, the release of presynaptic dopamine in the brain region through the D1R-mediated signaling is considered to be a more important pathway in the regulation of mental activity^18, 51, 52^. The G-protein-dependent cAMP/PKA/DARPP32 dopamine pathway has been well described to have multiple physiological responses, which is also referred to as slow synaptic transmission^53^. Interestingly, our results found that the pmTOR (Ser 2448) level was elevated by 10-day administration of *l*-PSD in naïve animals (Figure S2). Moreover, studies *in vitro* further confirmed that the increased pmTOR (Ser 2448) level was mediated by the PKA activation, while the effect was blocked by the PKA inhibitor H89 (Fig. 5). Several studies showed that ERK1/2 and PKB/AKT are linked to the activation of mTOR signaling by increasing phosphorylation level of pmTOR (Ser 2448) in depression^54^. In this work, we further identified the role of PKA as an upstream kinase to mTOR^55, 56^. Our present results indicated that the deficit of mTOR signaling in the mPFC may be due to a decrease of D1R/PKA signaling in the mPFC in depression.

Dysregulation of synaptic plasticity in the mPFC has been involved in the pathophysiology of depression^57–59^. Substantial studies had provided evidences regarding the negative effects of chronic stress on the synaptic and structural plasticity. Previous studies have shown that chronic stress induces impairment of LTP^60^ and retraction of apical dendritic arbors in the mPFC^61^. Current studies implied that the impaired synaptic and structural plasticity may be mediated by the inhibition of mTOR signaling in depression. mTOR is a regulator for the initiation of protein translation and synthesis. Elevated mTOR signaling promotes the synthesis of synaptic proteins that are necessary for synapse formation and maturation^35, 62^. In MDD patients, mTOR signaling is robustly down regulated in the mPFC^63^. CMS stimulation on animal models induced a deficit of mTOR signaling in the amygdala^64^. More importantly, the mTOR signaling pathway mediated rapid antidepressant response of ketamine (an NMDA receptor antagonist) and was regarded as a potential novel therapeutic target for antidepressant drug development^35^. The mTOR-dependent translational cascade in synaptic plasticity may be the final pathway that represents a rapid-acting and long-lasting onset of therapeutic benefit from antidepressants.

Our present results showed that CMS stimulation induced a significant decrease of pmTOR (Ser 2448) and synaptic-related proteins, such as PSD 95 and synapsin I in the mPFC, which was reversed by *l*-SPD administration (Fig. 3). Moreover, *l*-SPD triggered a long-term enhancement of synaptic transmission within 10–20 minutes treatment, which was blocked by the inhibition of D1R, PKA and mTOR (Fig. 5). Collectively, these results further indicate that the activation of D1R/AC/cAMP/PKA/mTOR cascade is essential for the *l*-SPD enhanced synaptic plasticity in the mPFC, which may be the mechanism of *l*-SPD for a rapid-acting onset of antidepressant effect.

**Figure 6:**
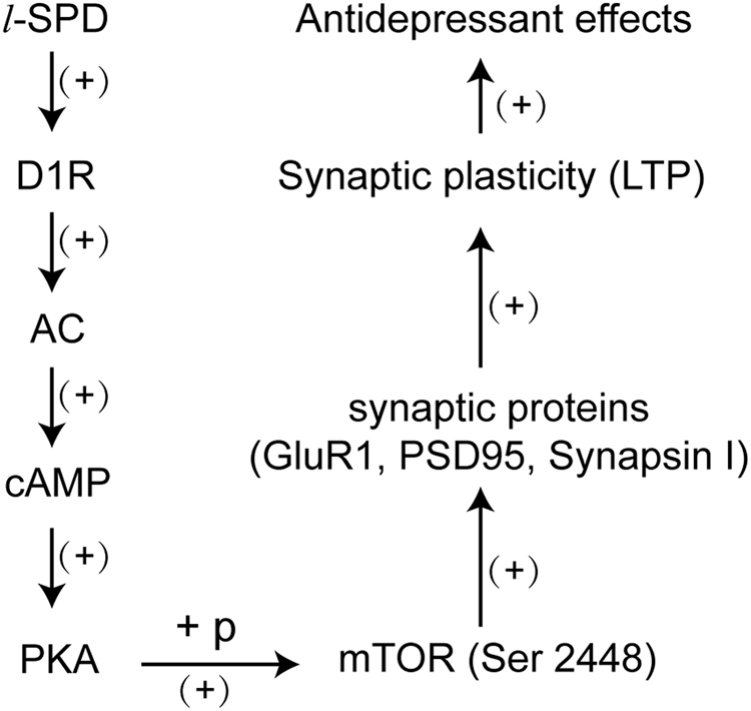
Proposed molecular mechanism of antidepressant effects of *l*-SPD. Through D1R agonism in mPFC, *l*-SPD activates AC/cAMP/PKA signaling. The activated PKA further increases phosphorylation of mTOR protein and activates mTOR signaling. The activated mTOR signaling enhances the rate of synaptic proteins, including PSD 95, synapsin I and GluR1, and then promoting synaptic plasticity.

In conclusion, the present study investigates the mechanism underlying the fast antidepressant-like effects of *l*-SPD. Using multiple approaches, our results verified that the D1R agonism of *l*-SPD led to an activation of D1R/cAMP/PKA/mTOR cascade in the mPFC, which subsequently produced a long-term enhancement of synaptic plasticity (Fig. 6). The present findings may provide new insights into the mPFC dopaminergic system and mTOR signaling in the pathophysiology of depression and shed light on the development strategies of new antidepressants.

## Methods

### Animals

Fifty male Sprague-Dawley rats aged 6 weeks, weighing 202 ± 1.5 g, were purchased from Shanghai Sippr-BK Laboratory Animal Co. Ltd. All animals were raised on a 12 hours dark/light cycle with 3–4 rats in one cage under specific pathogen free (SPF) condition. All procedures were carried out in accordance with the EU Directive 2010/63/EU on the protection of animals used for scientific purpose and the protocols were approved by the Animal Care Committees of Shanghai Institute of Materia Medica, Chinese Academy of Science.

### CMS procedure

An animal model of depression induced by chronic unpredictable mild stress, described in our previous report^43^, was used as a naturalistic rodent model of depression with slight modifications. Briefly, Sprague-Dawley rats were raised in separated cages and exposed to mild unpredictable stressors with different orders. The stressors including cold swimming for 5 min; isolated housing (one rat per cage); food or water deprivation for 12 hours; soiled cage; cage rotation for 12 hours; reversed dark/light cycle; white noise (65 dB SPL) for 12 hours. After 4-week induction of stressors, approximately 70% animals exhibited depressive- and anxiety-like phenotypes. The model animals were then divided into four groups (7–8 rats per group): (1) vehicle, (2) 10 mg·kg^−1^ Fluoxetine (FLX), (3) 5 mg·kg^−1^ *l*-SPD, and (4) 10 mg·kg^−1^ *l*-SPD administration.

### Forced swim test (FST)

The forced swim test (FST) was performed as previously described with slight modification^30^. Briefly, the Sprague-Dawley rats were placed in a transparent cylindrical bucket (height: 60 cm, diameter: 30 cm) which was filled with water (22–25 °C) to a depth of 45 cm. Rats were allowed to swim for 6 min and testes were repeated once a week, and their activity was videotaped. The duration of immobility defined as floating or remaining motionless was assessed. Due to the fact that most rats were very active for the initial 2 minutes and the potential effects of the treatment can be obscured during this period, the immobility of the last 4 min was analyzed by an observer blind of the animal treatments.

### Elevated plus maze (EPM)

Elevated plus maze (EPM) was performed the guides of a *Nat Protoc* ^31^. For Sprague-Dawley rats, the size of four arms was 50 × 10 cm with a central square (10 × 10 cm), and the wall of two closed arms were at the height of 50 cm with a height of 70 cm to the floor. At the beginning of the EPM test, the rats were comforted and placed in the center of maze with the head facing an enclosed arm. Each animal was allowed to perform for 6 minutes, tests were repeated every one week, and the activity was monitored online. Behaviors scores were calculated as the percent of the distance moved into the open arms. Arm entries were defined as entry of all four paws into the arm.

### Open field test (OFT)

Open field test (OFT) is a standard method to profile the locomotor activity. We performed this test as previous described^32, 33^. Briefly, the Sprague-Dawley rats were comforted and placed in the center of a square box (100 × 100 × 45 cm) that was made of black acrylic plastic. The rats were allowed to explore in the box for 10 min and were monitored online. The velocity for each animal was calculated offline.

### Primary cortical cell culture and drug treatment

Pregnant Sprague-Dawley rats with 17-day embryonic stage were anesthetized with isoflurane (inhal.) and then euthanized by cervical dislocation. The embryos were quickly removed, and the cerebral cortices were isolated under the microscope. The cortical tissue was cut with scissors and then digested in 0.125% trypsin at 37 °C for 15 min. The cell cluster was dispersed, and cells were plated on poly L-lysine-coated 6 well culture cluster (Corning). After 4-hour incubation in DMEM/HIGH GLUCOSE (HyClone) medium, the medium was replaced with Neurobasal medium (Gibco) containing B27 and glutamine. The cells were incubated under normal growth conditions (37 °C and 5% CO_2_), and the neurobasal medium was changed every 3 days.

### Slice preparation

Postnatal 16–21 days Sprague-Dawley rats were deeply anesthetized. Immediately after decapitation, the brain tissue was obtained. Bilaterally coronal mPFC (400-µm thick) was cut with a vibratome (Leica VT1000s, USA) in oxygenated (5% CO_2_, 95% O_2_) ice-cold modified artificial cerebrospinal fluid (mACSF) containing (in mM): 25.0 NaHCO_3_, 1.25 NaH_2_PO_4_, 2.5 KCl, 0.5 CaCl_2_, 7.0 MgCl_2_, 25.0 glucose, 11.0 choline chloride, 11.6 ascorbic acid and 3.1 pyruvic acid. The cut slices were quickly placed into a chamber and incubated in normal oxygenated ACSF (118 NaCl, 2.5 KCl, 26 NaHCO_3_, 1 NaH_2_PO_4_, 10 glucose, 1.3 MgCl_2_ and 2.5 CaCl_2_ in mM, gassed with 95% O_2_ and 5% CO_2_) at 32 °C for 1 h. The slice was finally transferred to the recording chamber at room temperature.

### Electrophysiology

Field excitatory postsynaptic potentials (fEPSP) were recorded using a Multiclamp 700B amplifier (Molecular Devices, USA) under a microscope (Olympus, Japan). For fEPSP recording, the bipolar stimulating electrode was placed in layer VI of the mPFC and the recording electrode (1–5 MΩ, filled with ACSF) was placed in layer II/III. The data were filtered at 2 kHz, sampled at 10 kHz using a Digidata 1440 A (Molecular Devices, USA) and analyzed with Clampfit 10.2 software (Molecular Devices, USA). The duration of the stimuli pulse was 100 µs delivered at 0.03 Hz using an electronic stimulator (Nihon Kohden Corporation, Japan), and the stimuli intensity was adjusted to 65% of the maximal response. The evoked response was monitored for 10 minutes at this intensity to ensure stable recording prior to the drug treatment. All recordings were continued for 60–80 minutes.

### Drug administration

For the *in vivo* experiments, *l*-SPD was dissolved into 0.1 M H_2_SO_4_ with pH 5.0–5.5, and FLX was dissolved into ddH_2_O. *l*-SPD (5 mg·kg^−1^ and 10 mg·kg^−1^), FLX (10 mg·kg^−1^) and vehicle (0.1 M H_2_SO_4_ solution with pH 5.0–5.5) were intraperitoneally (i.p.) administered once daily at 9:00 am for 3 weeks. The injected volume was 5 ml/kg. For the *in vitro* experiments, *l*-SPD was also dissolved in 0.1 M H_2_SO_4_ with pH: 5.0–5.5. SCH23390 and Quinpirole were dissolved into ddH_2_O. H89 and rapamycin were dissolved in DMSO. In all experiments, the final concentration of DMSO was no more than 0.1%. *l*-SPD was purchased from Ji Qi Pharmaceutical Company, Guilin, China, 541004. Fluoxetine and other chemicals were purchased from Sigma-Aldrich, USA.

### Western blot

The rodents were deeply anesthetized and euthanized by decapitation. The bilateral PFC regions were dissected out immediately and homogenized by sonic disruption. For cultured primary cells, after 4 hours drug treatment, the cells were harvested. The next steps for PFC tissues and primary cells were the same. The whole homogenate or cell lysate was centrifuged at 14,000 × *g* for 10 min, and the total protein concentration was assessed. The proteins were separated on SDS-PAGE gel and transferred to a nitrocellulose membrane. The membrane was blocked and then incubated with primary antibody (anti-mTOR, 1:1000, Cell Signaling; anti-pmTOR (Ser 2448), 1:1000, Cell Signaling; anti-pPKA Cα (Thr197), 1:1000, Cell Signaling; anti-PKA Cα, 1:1000, Cell Signaling; anti-PSD 95, 1:1000, Invitrogen; anti-GluR1, 1:1000, Abcam; anti-synapsin I, 1:1000, Cell Signaling; or anti-tubulin, 1:1000, Cell Signaling) at 4 °C overnight. The membrane was washed and incubated with secondary antibody (goat anti-Rb IgG, 1:5000, Abcam). The membrane was washed, chemiluminescence (ECL) reaction solution was added, and then the samples were exposed to X-ray film (Carestream) for visualization.

### Statistical analysis

The data are expressed as the mean ± SEM. The results of open field test in Sprague-Dawley rats were analyzed using one-way ANOVA, and post hoc LSD tests were performed. The results of forced swim test and elevated plus maze in Sprague-Dawley rats were analyzed using repeated ANOVA and post hoc LSD tests were performed. The western blot results were analyzed using one-way ANOVA, and post hoc LSD tests were performed. For fEPSP results, paired *t*-tests were performed to analyze the slope differences of fEPSP between baseline and *l*-SPD/vehicle treatment.

## Electronic supplementary material

Supplementary Information
