# Supplementary material for: Activation of D1R/PKA/mTOR signaling cascade in medial prefrontal cortex underlying the antidepressant effects of l-SPD
Source: Sci Rep. 2017 Jun 19;7:3809. doi: 10.1038/s41598-017-03680-2 (PMC5476681; doi:10.1038/s41598-017-03680-2)
Supplement: Supplementary file 1 — Supplementary Information [file 41598_2017_3680_MOESM1_ESM.pdf]

Supplemental information

Activation of D1R/PKA/mTOR signaling cascade in medial prefrontal cortex underlying the antidepressant effects of *l*-SPD

Bing Zhang[1,2], Fei Guo[1]*, Yuqin Ma[1,2], Yingcai Song[3], Rong Lin[3], Fu-Yi Shen[3], Guo-Zhang Jin[1#], Yang Li[1#],Zhi-Qiang Liu[3#]*

Supplemental methods

Animals

Thirty-eight male C57BL/6 mice aged 8 weeks, weighing 25.1 ± 1.5 g, were purchased from Shanghai Sippr-BK Laboratory Animal Co. Ltd. All animals were raised on a 12 hours dark/light cycle with 5 mice in one cage under specific pathogen free (SPF) condition. All procedures were carried out in accordance with the EU Directive 2010/63/EU on the protection of animals used for scientific purpose and the protocols were approved by the Animal Care Committees of Shanghai Institute of Materia Medica, Chinese Academy of Science. The naïve C57BL/6 mice were divided into four groups (9-10 mice per group): 1) vehicle, 2) 5 mg·kg^{-1} *l*-SPD, 3) 10 mg·kg^{-1} *l*-SPD and 4) 10 mg·kg^{-1} fluoxetine administration (i.p.). All drugs were administrated once daily at 9:00 am for 10 days. The injected volume was 10 ml/kg

Tail suspension test (TST)

Tail suspension test (TST) is the most commonly used experiments to assess depressive-like behavior [1]. In this experiment, naïve C57BL/6 mice were moved to the testing room 2 h before the behavioral tests started. The mice were suspended by fixing their tail on a TST apparatus. The test lasted 6 min, and the process was monitored online. Due to the most mice were very active at the beginning of the test and the potential effects of the treatment can be obscured during the first two minutes, the immobility of the last 4 min were measured.

Elevated plus maze (EPM)

The EPM apparatus for C57BL/6 mice consisted of two enclosed (30 x 5 x 30 cm) and two open arms (30 x 5 cm) with a height of 60 cm to the floor. The same type of arm was opposite to each other and connected by a central area (5 x 5 cm). At the beginning of the EPM test, the mice were comforted and placed in the center of maze with the head facing an enclosed arm. Each animal was allowed to perform for 6 minutes, and the activity was monitored online. Behaviors scores were calculated as the percent of the distance moved into the open arms. Arm entries were defined as entry of all four paws into the arm.

Immunohistochemistry (IHC)

The C57BL/6 mice were deeply anesthetized and perfused with 0.9% physiological saline. Next, the mice were perfused with 4% paraformaldehyde and immersed into 4% paraformaldehyde for 24 hours. The brain was dehydrated in 30% sucrose for 3 days. For the location of the PFC, we referred to the mouse brain atlas and the thickness of brain slice was 30 μm. Brain slices were treated by 0.3% Triton X-100 and 0.3% H_2O_2 and then blocked in 10% goat serum (Gibco) and incubated in primary antibody (anti-pmTOR (Ser 2448), 1:50, Cell Signaling; anti-PSD 95, 1:50, Invitrogen) at 4 ℃ overnight. Brain slices were washed and incubated in secondary antibody (goat pAb to Rb IgG, 1:200, Abcam) for 2 hours. Then, the brain slices were incubated in streptavidin biotin peroxidase complex (SABC) for 1 hour and then washed and transferred to microscope slides. After being dried, the brain slices were stained using DAB substrate solution for 5 min. Brain slices were dehydrated in gradient alcohol (70%, 95%, 100% and 100%) and cleared by xylene. Mounted brain slice by mounting solution and observed under a microscope.

Statistical analysis

The data are expressed as the mean ± SEM. The results of behavioral tests including TST and EPM in C57BL/6 mice were analyzed using one-way ANOVA, and post hoc LSD tests were performed. The western blot results were analyzed using one-way ANOVA, and post hoc LSD tests were performed.

Reference

1. Steru, L., Chermat, R., Thierry, B. & Simon, P. The Tail Suspension Test - a New Method for Screening Antidepressants in Mice. *Psychopharmacology* **85**, 367-370 (1985).

Supplemental figures and figure legends

Figure S1

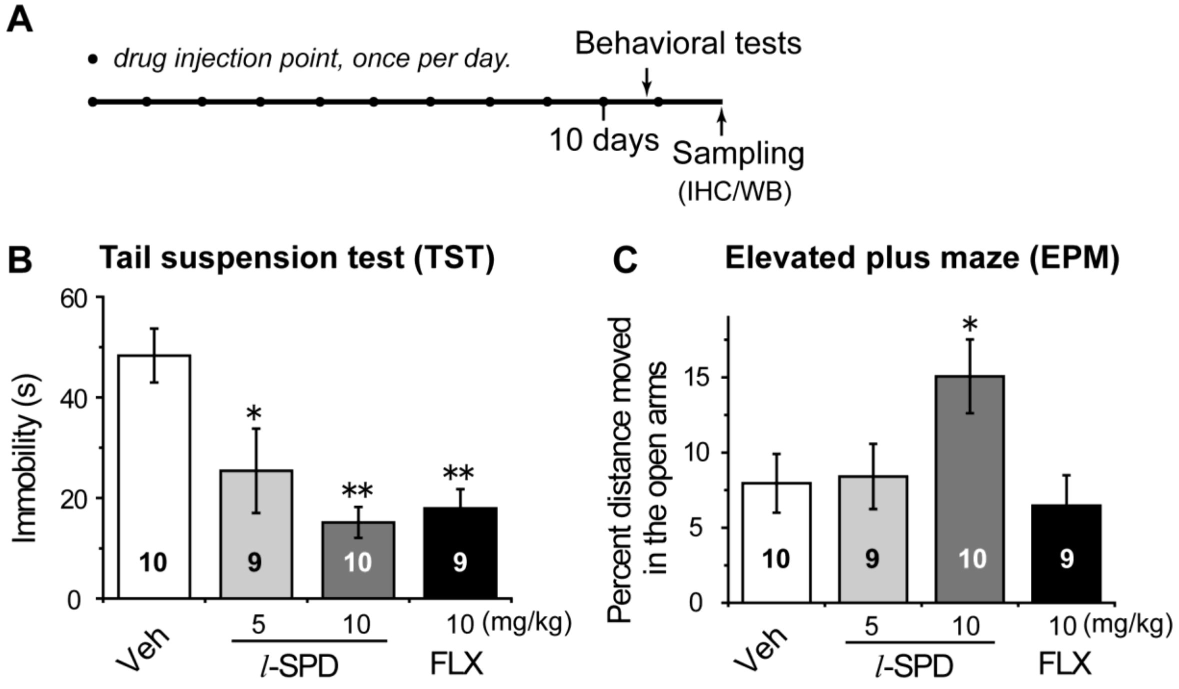

Figure S1

Antidepressant-like and anxiolytic-like effects of *l*-SPD in C57BL/6 mice. (A) The schematic representation for experimental procedures. The C57BL/6 mice were treated with vehicle, *l*-SPD (5 mg ·kg^{-1} or 10 mg ·kg^{-1}) and fluoxetine (10 mg ·kg^{-1}) daily for 10 days, the drugs were administrated at 9:00 am every day. Behavioral tests were performed 24 hours after the last drug injection. The brain tissues were obtained 24 hours after the last drug administration. (B) Tail suspension test: the immobility time of mice were measured in the tail suspension test. (C) Elevated plus maze: the percent distance in the open arms was measured in the elevated plus maze. The inserted numbers represent the number of animals in each experimental group. *$p < 0.05$, **$p < 0.01$, compared to the vehicle-treated group.

Figure S2

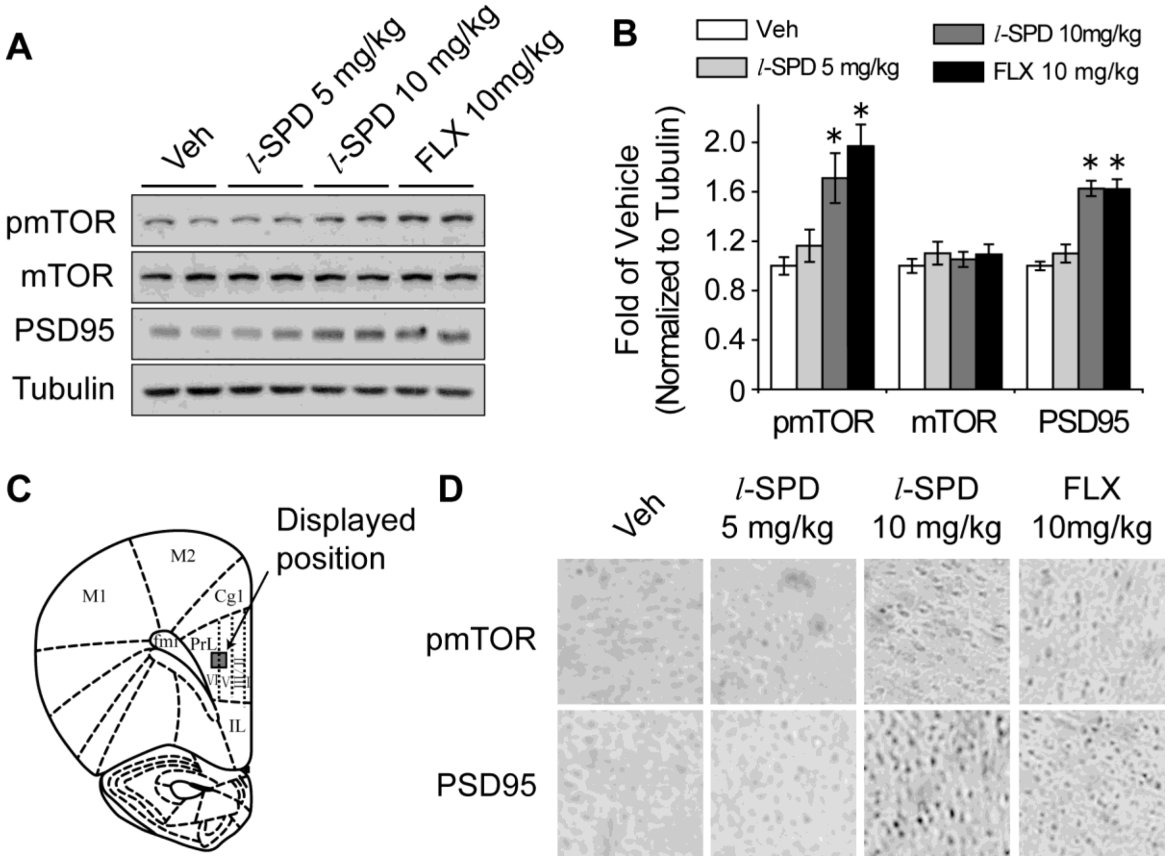

Figure S2

***l*-SPD enhanced mTOR signaling and synaptic protein expression of mPFC in C57BL/6 mice.**

The mPFC tissues of C57BL/6 mice were obtained after behavioral tests were completed. (A) Western blot analysis of pmTOR (Ser 2448), mTOR, PSD 95 and beta-tubulin. (B) Quantification analysis of protein levels of pmTOR (Ser 2448), mTOR, PSD 95 shown in panel A, β-tubulin expression levels were determined as the control. (C) A schematic diagram of coronal mPFC. The small square represents the displayed position shown in panel D. (D) Immunohistochemistry analysis of pmTOR (Ser 2448) and PSD 95 in the layer V-VI of mPFC. *$p < 0.05$, compared to the vehicle treatment group.

Figure S3

Figure 3A

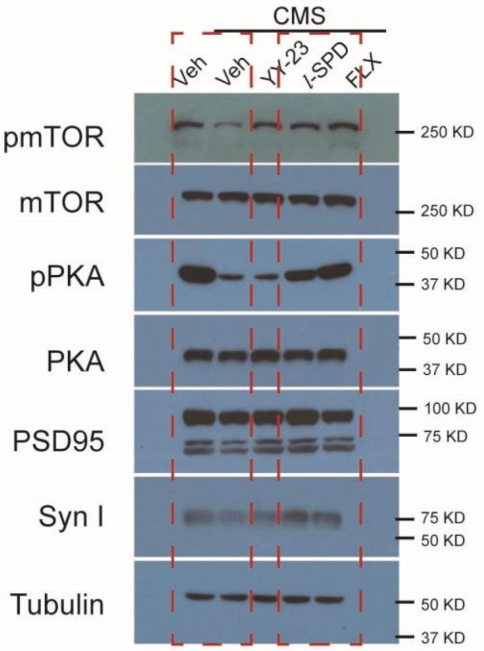

Figure 4A, B, C

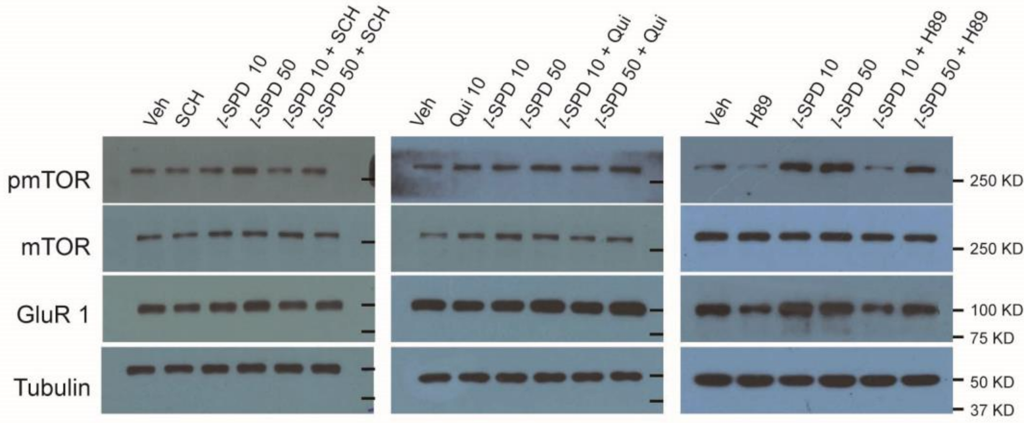

Figure S2A

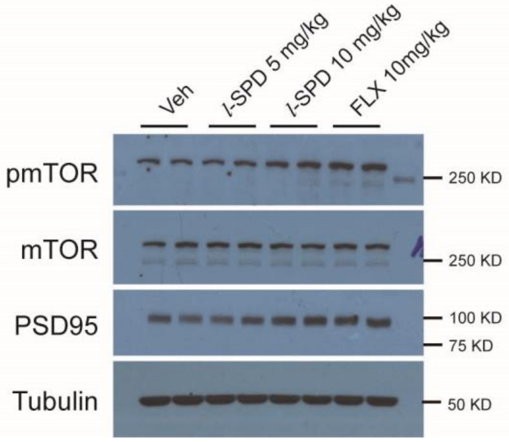

Figure S3

Full-length blots corresponding to the results showed in Figure 3A, Figure 4A-4C and Figure S2A. In Figure 3A, only red dashed boxes part were displayed.
